# Policy and Environmental Indicators for Heart Disease and Stroke Prevention: Data Sources in Two States

**Published:** 2004-03-15

**Authors:** Delores M. Pluto, Martha M. Phillips, Dyann Matson-Koffman, Dennis M. Shepard, James M. Raczynski, J. Nell Brownstein

**Affiliations:** Prevention Research Center, Arnold School of Public Health; Department of Epidemiology, School of Public Health, University of Alabama at Birmingham (presently with the Dept. of Psychiatry and Behavioral Sciences, Centers for Mental Healthcare Research, University of Arkansas for Medical Sciences); Cardiovascular Health Branch, Division of Adult and Community Health, National Center for Chronic Disease Prevention and Health Promotion, CDC; Prevention Research Center, Arnold School of Public Health, University of South Carolina; Center for Health Promotion and Department of Health Behavior, School of Public Health, University of Alabama at Birmingham (presently with the College of Public Health, University of Arkansas for Medical Sciences); Cardiovascular Health Branch, Division of Adult and Community Health, National Center for Chronic Disease Prevention and Health Promotion, CDC

## Abstract

**Introduction:**

Investigators in South Carolina and Alabama assessed the availability of data for measuring 31 policy and environmental indicators for heart disease and stroke prevention. The indicators were intended to determine policy and environmental support for adopting heart disease and stroke prevention guidelines and selected risk factors in 4 settings: community, school, work site, and health care.

**Methods:**

Research teams used literature searches and key informant interviews to explore the availability of data sources for each indicator. Investigators documented the following 5 qualities for each data source identified: 1) the degree to which the data fit the indicator; 2) the frequency and regularity with which data were collected; 3) the consistency of data collected across time; 4) the costs (time, money, personnel) associated with data collection or access; and 5) the accessibility of data.

**Results:**

Among the 31 indicators, 11 (35%) have readily available data sources and 4 (13%) have sources that could provide partial measurement. Data sources are available for most indicators in the school setting and for tobacco control policies in all settings.

**Conclusion:**

Data sources for measuring policy and environmental indicators for heart disease and stroke prevention are limited in availability. Effort and resources are required to develop and implement mechanisms for collecting state and local data on policy and environmental indicators in different settings. The level of work needed to expand data sources is comparable to the extensive work already completed in the school setting and for tobacco control.

## Introduction

Beginning in 1998, the Centers for Disease Control and Prevention (CDC) received federal funding to support state heart disease and stroke prevention programs. The purpose of these state programs is to develop comprehensive programs emphasizing community-based policy and environmental strategies to reduce risk factors related to heart disease and stroke, such as physical inactivity, poor nutrition, tobacco use, and hypertension. The CDC recommends that assessment and policy development be included within the 10 core public health services to support individual and community health efforts. To monitor their progress on developing community-based policy and environmental strategies, state programs require intermediate evaluation measures of policy and environmental factors. Community-level indicators have been used to measure such intermediate policy and environmental outcomes for other community-based disease prevention programs (1,2). For example, community-level indicators for tobacco use include the existence and quality of clean air laws and the presence of cigarette vending machines in restaurants.

The Cardiovascular Health Branch of the CDC, in collaboration with other units within the National Center for Chronic Disease Prevention and Health Promotion, used literature searches, expert recommendations, and a Delphi process to identify policy and environmental indicators associated with physical activity, nutrition, tobacco control, and national heart disease and stroke prevention guidelines. A draft list of 31 pilot policy and environmental indicators was developed with the intention of revising the list upon feedback from this study. The indicators were selected, in part, because they were thought to be feasible for consistent measurement across 50 states. For example, one indicator can be used to track the number of states that have policies requiring daily physical education for grades K–12. The indicators were categorized by community, school, work site, or health care setting (3).

Because literature on community-level indicators was limited, little was known about the availability of data sources for use by state heart disease and stroke prevention programs. Hence, the Cardiovascular Health Branch staff asked the Alabama and South Carolina heart disease and stroke prevention program directors to assess the availability of data sources for the 31 pilot indicators in those 2 states and to provide their perspectives on the feasibility of using these indicators. These 2 states were selected because of their proximity to the CDC in Atlanta for technical assistance and because each state program has a close relationship with its Prevention Research Center. Each state program collaborated with its Prevention Research Center (the Center for Health Promotion at the University of Alabama at Birmingham and the Prevention Research Center at the University of South Carolina) to carry out the assessment. This paper summarizes the findings and provides recommendations for collecting data and refining community-level indicators for the surveillance of heart disease and stroke prevention.

## Methods

Between October 2000 and October 2001, research teams at the South Carolina and Alabama Prevention Research Centers worked in tandem to identify and examine possible data sources and to assess sensitivity and specificity for each indicator. To identify possible data sources, the research teams completed a systematic search within each of 4 settings: community, school, work site, and health care. They identified individuals in state departments of health and education, other state agencies, and private organizations who might have access to or be aware of relevant data sources (Table 1).

Individuals were identified using a snowball technique that began with people or organizations known to research team members as well as contacts identified from Web sites. As individuals were identified, a team member contacted them by telephone. A conversational interview was used to ask respondents if they collected any data related to a given indicator, and if so, they were asked to provide details about the data source. If the agency or organization did not collect relevant data, the research team requested names of other potential informants or sources of data. These new informants were contacted and the process was repeated until all identified individuals or agencies were contacted.

Additionally, the research teams completed literature and on-line searches using keywords from each indicator (e.g., sidewalks, mixed-use, bicycle) to identify additional data sources and possible contacts. Once data sources were identified, the research teams reviewed each data source, taking note of the degree to which the data fit the indicator; the frequency and regularity with which data were collected; the consistency of the data collected across time; the costs (time, money, personnel) associated with data collection and/or data access; and the accessibility of data.

In addition to evaluating the data sources, the research teams made a general assessment of the sensitivity and specificity of each indicator. Sensitivity refers to the extent to which an indicator allows for documentation of incremental change. Indicators were flagged as lacking sensitivity if they referred only to the presence or absence of a policy rather than the extent to which a policy addressed an issue. Indicators were also flagged as lacking sensitivity if they measured change at an inappropriate level (i.e., if an indicator asked about state policy when policy is set at the local level). Specificity refers to the extent to which an indicator precisely and accurately describes an environmental feature or policy being measured. Indicators were flagged as lacking specificity if they were ambiguous or failed to define key terms.

During this project, research teams participated in regular conference calls with personnel from the CDC's Cardiovascular Health Branch and the state program managers in Alabama and South Carolina to review progress, clarify issues, and share protocols and information. Although each research team completed tasks independently and had a different contractual relationship with its state program, efforts were made to ensure that working protocols (including evaluation criteria and reporting formats) were consistent.

## Results

Among the 31 pilot indicators, 11 (35%) had readily available data sources and 4 (13%) had data sources that could provide at least partial measurement. Data sources were available for most indicators in the school setting and for indicators related to tobacco policies across all settings. Data sources were least available in the work site and health care settings. Most data sources identified were maintained by a national agency or organization (e.g., CDC, U.S. Department of Agriculture [USDA], National Transportation Enhancements Clearinghouse). State agencies often report data to these national data sources. Neither research team found a data source unique to its state.

The list of indicators was in draft form at the time of this assessment; thus, many pilot indicators were found to lack specificity. Ten (37%) indicators were flagged as lacking specificity because of ambiguous or imprecise definitions. In addition, 9 (29%) indicators were flagged as lacking sensitivity because they considered only the presence or absence of state legislation, not the quality or degree to which recommendations were included in the legislation. More detailed results are presented about the data sources found in each of the 4 settings.

### Community setting

Two of the 8 pilot indicators in the community setting — clean indoor air laws and smoking in the home — have readily available data sources (Table 2).

The legislative database in the State Tobacco Activities Tracking and Evaluation (STATE) system summarizes state tobacco legislation, including smoke-free indoor air ordinances for restaurants, day care centers, and public places (4,5). The Office on Smoking and Health at the CDC maintains the database, based on a quarterly search of the LexisNexis legal database (4,5). The database can be used to monitor the presence or absence of state policies and the content of those policies (e.g., restrictions, penalties, enforcement). The legislative database, however, does not capture municipal ordinances that might be enacted in the absence of state policies. Beginning in 1998, the optional Tobacco Indicators module of the annual Behavioral Risk Factor Surveillance System (BRFSS) asked respondents if anyone smoked anywhere in their homes. In 2001, this was changed to ask if smoking was allowed in their homes (6). The Tobacco Indicators module was used by 25 states in 2002.

Data sources also are available that partially measure 2 other community indicators: highway funding of transportation alternatives and the number of farmers' markets. The National Transportation Enhancements Clearinghouse maintains a database of transportation enhancements funds allocated and spent by each state under the Transportation Equity Act for the 21st Century (TEA-21). This searchable, on-line database is updated annually (7). Funds for transportation alternatives under TEA-21, however, do not represent the entire state budget for transportation alternatives, and the database does not include the total amount of the state transportation budget. The research teams found no additional data sources that provide relevant details on highway spending at the state or local level.

The USDA maintains a list of farmers' markets searchable on-line by state (8). The database depends on reports from individual state departments of agriculture. Because the definition of a farmers' market varies by state, the data might be inconsistent or incomplete across states. For example, at the time of this study, the South Carolina listing included only 3 state-run, year-round farmers' markets. The list was recently updated to include smaller local markets that operate on a seasonal basis.

Although regional milk production figures are available, no state data were found on milk production or sales. The research teams also noted that this indicator is not a measure of environment or policy but a community-level indicator of purchasing behavior.

### School setting

Ten pilot indicators for heart disease and stroke prevention were identified in the school setting (Table 3). Seven indicators that refer to state policies on physical education requirements, student physical education assessments, food availability, certifications for food service staff and physical and health education teachers, and health education curriculum have readily available data sources.

All 7 of these indicators can be assessed using data from the School Health Policies and Programs Study (SHPPS), which is conducted every 6 years. The study surveys all state departments of education and a nationally representative sample of districts and schools (11). The state survey includes questions related to each of the 7 school indicators. These indicators assume that such policies are enacted at the state level; however, in states like South Carolina and Alabama, school policies are under the authority of school districts or the schools themselves.

The School Health Education Profile (SHEP) collects data that provide partial measurement of school health councils and tobacco-free schools. SHEP is a survey completed every 2 years by a sample of school principals and lead health educators in public schools containing classrooms at the sixth-grade level or higher (12). Because no similar data source is available for elementary schools, SHEP can only partially measure these indicators. In addition, the survey does not currently include questions that lead to the assessment of all components of the tobacco-free school policies recommended by the CDC.

### Work site setting

Only one of the 8 pilot work site indicators — clean indoor air laws for work sites — has a readily available data source (Table 4). Neither research team found any data sources for other work site indicators.

The STATE system contains information that measures state clean air laws that apply to work sites (4,5). This indicator is subject to the same sensitivity concerns previously noted for other clean indoor air laws — it notes only the presence or absence of state policies. The BRFSS optional Tobacco Indicators module collects information from individuals about their work site tobacco policies, but it does not measure state indoor air laws. Data on work site policies collected by the optional module would provide an estimate of the percentage of employed adults protected by a work site smoking policy.

Questions from the National Worksite Health Promotion Survey could be used to assess on-site physical activity programs and nutrition or weight management programs (13). This survey collects and provides national data for *Healthy People 2010* (14). The sample is too small, however, to draw conclusions by state. Other measurement tools assess policies and environmental characteristics related to heart disease and stroke prevention within work sites, including *Heart Check* (15) and the *Checklist of Health Promotion Environments at Worksites* (16). However, these instruments are not commonly used across the country and are not designed to be used as surveillance tools.

### Health care setting

Among the 5 pilot indicators identified in the health care setting, only one has a readily available data source: smoking cessation advice delivered by health care professionals (Table 5). The proportion of smokers who received advice to quit smoking in the past year has been included in the optional Tobacco Indicators module of the BRFSS since 2000.

## Discussion

In Alabama and South Carolina, the school setting has data to measure — at least partially — all but one of the pilot indicators for heart disease and stroke prevention. The community, work site, and health care settings have data sources for fewer than half of the indicators.

### Improving data collection

Given the overall lack of data in most settings assessed in this study, consideration should be given to designing and implementing new data collection processes. Vehicles for new data collection efforts are likely to be surveillance efforts now supported by the CDC (e.g., BRFSS, Youth Risk Behavior Surveillance System, SHPPS, SHEP). The SHPPS and SHEP are designed to collect policy data and are updated regularly to include more complete information. For example, SHEP 2002 included questions related to 2 school indicators: the percent of schools that provide health education instruction that includes the physical education topics listed in CDC's School Health Index and the proportion of schools that have adopted tobacco-free policies that meet CDC recommendations (20,21). Although the BRFSS is an individual-level surveillance tool, the optional Tobacco Indicators module already allows states to collect data to measure 2 indicators indirectly (smoking in the home and receiving advice to quit). Because this module is optional, the data are not available in all states. The availability and variability of relevant data across states can have important implications for achieving consistency within a national surveillance system for heart disease and stroke prevention. This study, however, did not explore a sufficient number of states to determine the extent of this variability.

Systems similar to the legislative database of the STATE system could be developed to monitor other state policies. In fact, in late 2003, the CDC Division of Nutrition and Physical Activity launched an on-line searchable database containing bill information related to physical activity and nutrition from all 50 states (22). Few existing national surveillance efforts, however, gather information from local governments, work sites, and health insurers. Important issues of cost — in terms of time, personnel, financial resources, and participant burden — must be considered when developing new data collection efforts or revising existing systems.

Although the research teams made extensive efforts to consult with a wide range of organizations, other data sources might exist. The research teams restricted their exploration to data that are collected either nationally or within their states. While this project did not complete an exhaustive review of data sources in other states, it did identify some noteworthy examples, such as New York's Heart Check (15). Additional surveys developed by other states (e.g., Montana, North Carolina) can be found on the Cardiovascular Health Council of the Chronic Disease Directors Web site: http://www.chronicdisease.org/cvh_council/Key%20Elements/ State%20Survey/CVH_state_survey.htm. The mechanisms illustrated at this site can serve as models for other states.

An additional challenge of data collection is assessing the impact of policy and environmental changes on behavior and health. Policy and environmental indicators provide only one part of the equation. For example, assessing the impact of school policies on children's behavior presents challenges in obtaining informed consent from the children, school administration, and/or parents.

### Refining indicators

To be useful to state programs, indicators for heart disease and stroke prevention examined in this study need to be refined to improve specificity and sensitivity. Including clear definitions would improve the specificity of the indicator and the accuracy and consistency of data collected. Sensitivity for many indicators could be enhanced by establishing criteria for evaluating policies and laws beyond consideration of their presence or absence at the state level. Some data sources like STATE and SHPPS already collect detailed information that could be used to evaluate the content and quality of policies in addition to tracking their presence or absence.

While it may be sufficient to look at states' policies for national surveillance, state programs might need additional surveillance data that show progress in meeting prevention goals within their own states. In some cases, particularly within school and community settings, it might be more relevant — albeit more costly — to assess the percentage of local jurisdictions (counties, municipalities, school districts) that implement a given policy.

The health care indicators provide the greatest challenge for surveillance. As worded, the indicators look at the percentage of insurers that provide a specific type of coverage. Knowing this information might not reflect the percentage of the population covered by those companies. For example, South Carolina currently has only 5 health maintenance organizations, which cover less than 10% of the state's population (23). Even if data indicated that all of these organizations followed the recommended guidelines, the data would not include 90% of the South Carolinians who might or might not have coverage under some other type of health care plan. In addition, insurance companies tend to negotiate with individual employers about the content of health insurance plans rather than having standard plans. Nationally, employers provide coverage for 58% of the population (23). If employer surveys are developed for other work site indicators, these surveys could include questions about health insurance provided by the employers.

The results of this investigation support the need for more attention, resources, and research to provide a consistent, documentable system for measuring indicators for heart disease and stroke prevention. It also will be important to improve the sensitivity and specificity of each indicator and to evaluate how each indicator corresponds to risk factors and health outcomes. These recommendations are consistent with the new *Public Health Action Plan to Prevent Heart Disease and Stroke*, which recommends enhancing data sources and systems to monitor key indicators for heart disease and stroke and "to systematically evaluate policy and program interventions" (24). Currently, the CDC is funding other projects to refine and validate these and other potential indicators for heart disease and stroke. With the evolving importance of policy and environmental factors influencing primary and secondary prevention efforts in public health, it is vital that a system be developed that will provide national, state, and possibly local data on indicators for heart disease and stroke. During the next decade, these indicators could provide valuable measurements to determine how environmental and policy changes are affecting heart disease and stroke prevention in this nation.

## Figures and Tables

**Table 1:** Examples of Agencies and Organizations Contacted for Information on Data Sources for Heart Disease and Stroke Prevention, South Carolina and Alabama, 2001

**Setting**	**Agency or Organization**
**Community**	Federal and state departments of transportation State and local departments of parks and recreation Federal and state departments of agriculture National Transportation Enhancements Clearinghouse Associations of mayors State and national dairy associations CDC Office on Smoking and Health CDC Behavioral Surveillance Branch
**School**	State departments of education CDC Division of Adolescent and School Health
**Work site**	Better Business Bureau Local work site wellness associations CDC Division of Adult and Community Health
**Health care**	State insurance commissioners Major third-party insurers (e.g., Blue Cross/Blue Shield, health maintenance organizations)

**Table 2:** Pilot Indicators and Data Sources for Heart Disease and Stroke Prevention, Community Setting, South Carolina and Alabama, 2001

**Indicator**		**Data Sources and Comments**	
1.	Percent of highway funds devoted to transportation alternatives (e.g., bicycle lanes linked to public transportation, mass transit systems, facilities and roadway changes; supports such as parking hubs and bicycle racks).^a^	1.	National Transportation Enhancements Clearinghouse (http://www.enhancements.org). Includes only data on funding spent under the federal Transportation Enhancements Program.^c^
2.	Percent of counties or municipalities with policies requiring sidewalks in all new and redeveloped residential and mixed-use communities.	2.	No data source found.
3.	Percent of counties or municipalities with policies that promote recreation facilities (e.g., bikeways, parks, fields, gyms, pools, tennis courts, and playgrounds) in new and redeveloped residential and mixed-use communities.	3.	No data source found.
4.	State policies and percent of counties or municipalities with policies and strategic plans to promote bicycle use for transportation purposes.	4.	No data source found.
5.	Percent of low-fat milk sales in the state (1= or less).	5.	No data source found. Regional milk production data are available but do not reflect state sales.
6.	Number of farmers' markets per capita in the state.^a^	6.	U.S. Dept. of Agriculture Farmers' Market database (http://www.ams.usda.gov/ farmersmarkets/). Incomplete due to inconsistent reporting and definition of farmers' markets across states.^c^
7.	State with laws on smoke-free indoor air that prohibit smoking or limit it to separately ventilated areas in restaurants, day care centers, and other public places.^b^	7.	State Tobacco Activities Tracking and Evaluation (STATE) System (http://www2a.cdc.gov/ nccdphp/osh/state/).^d^
8.	Proportion of smokers who report that smoking is not allowed anywhere inside their homes.	8.	Behavioral Risk Factor Surveillance System (BRFSS), optional Tobacco Indicators module (http://www.cdc.gov/brfss).^d^

a 2 indicators (25%) lack specificity (ambiguous, lack precision).

b 1 indicator (12%) lacks sensitivity (unable to measure incremental change, measured at inappropriate level).

c 2 indicators (25%) have data sources that partially measure indicator.

d 2 indicators (25%) have adequate data sources.

**Table 3:** Pilot Indicators and Data Sources for Heart Disease and Stroke Prevention, School Setting, South Carolina and Alabama, 2001

**Indicator**		**Data Sources and Comments**	
1.	State policies that require daily physical education or its equivalent in minutes per week, for all students in K–12, with no substitution of other courses or activities for physical education.^a^	1.	School Health Policy and Programs Study (SHPPS) (www.cdc.gov/ nccdphp/dash/shpps).^c^
2.	State policies that require schools to assess students on the knowledge and skills specified by the state's physical education standards, frameworks, or guidelines.^a^	2.	SHPPS.^c^
3.	State policies requiring that the foods and beverages available at schools outside of school meal programs reinforce the principles of the *Dietary Guidelines for Americans* (9).^a^	3.	SHPPS.^c^
4.	State policies that require newly hired school food service managers to have a nutrition-related baccalaureate or graduate degree and certification/credentialing in food service from either the state or the American School Food Service Association.^a^	4.	SHPPS.^c^
5.	State policies that require all newly hired staff who teach physical education to be certified, licensed, or endorsed by the state to teach physical education.^a^	5.	SHPPS.^c^
6.	State policies that require all newly hired staff who teach health education to be certified, licensed, or endorsed by the state to teach health education.^a^	6.	SHPPS.^c^
7.	States policies that require schools to assess students on the knowledge and skills specified by the state's health education standards, frameworks, or guidelines.^a^	7.	SHPPS.^c^
8.	Percent of schools that provide health education instruction that includes the physical education, nutrition, and tobacco use prevention topics listed in *School Health Index* (10).	8.	No data source found. Questions from *School Health Index* could be useful for surveillance, if survey mechanism is developed.
9.	Proportion of schools with School Health Councils.^b^	9.	School Health Education Profile (SHEP) (http://www.cdc.gov/nccdphp/dash/profiles). SHEP is completed by sample of principals and lead health educators in schools having at least one of the grades 6–12. No data source available for elementary schools.^d^
10.	Proportion of schools that have adopted tobacco-free school policies that meet CDC recommendations.^b^	10.	SHEP. See 9 above. SHEP does not include questions to thoroughly assess if tobacco policies meet recommendations.^d^

a 7 indicators (70%) lack sensitivity (unable to measure incremental change, measured at inappropriate level).

b 2 indicators (20%) lack specificity (ambiguous, lack precision).

c 7 indicators (70%) have adequate data sources.

d 2 indicators (20%) have data source that could partially measure indicator

**Table 4:** Pilot Indicators and Data Sources for Heart Disease and Stroke Prevention, Work Site Setting, South Carolina and Alabama, 2001

**Indicator**		**Data Sources and Comments**	
1.	Percent of work sites that have policies supporting the engagement of all employees in physical activity during work time (e.g., flexible scheduling, relaxed dress codes).	1.	No data source found.
2.	Percent of work sites that provide showers and changing facilities to support physically active employees.	2.	No data source found.
3.	Percent of work sites that provide and promote on-going, on-site employee physical activity programs (e.g., walking, stretching, aerobics) during the previous 24 months.	3.	No data source found. National Worksite Health Promotion Survey measures this indicator at the national level, but the sample is too small for state analysis.
4.	Percent of work sites with vending machines and/or snack bars that offer heart-healthy food and beverage choices, including water or flavored water, 1% or less milk products, 100% juice products, fruits, vegetables, and products labeled low or reduced calorie, low or reduced sodium, and those labeled 3 grams or less of fat per serving.	4.	No data source found.
5.	Percent of work sites with cafeterias that offer heart-healthy food and beverage choices including water or flavored water, 1% or less milk products, 100% juice products, fruits, vegetables, and products labeled low or reduced calorie, low or reduced sodium, and those labeled 3 grams or less of fat per serving.	5.	No data source found.
6.	Percent of work sites that offer nutrition or weight management classes or counseling.^a^	6.	No data source found. National Worksite Health Promotion Survey measures this indicator at the national level, but the sample is too small for state analysis.
7.	States with laws on smoke-free indoor air that prohibit smoking or limit it to separately ventilated areas in government and private work sites.^b^	7.	State Tobacco Activities Tracking and Evaluation System (STATE) (http://www2a.cdc.gov/nccdphp/osh/state/").^c^
8.	Proportion of work sites (segmented by number of employees) that cover smoking cessation programs.^a^	8.	No data source found.

a Two indicators (25%) lack specificity (ambiguous, lack precision).

b One indicator (12%) lacks sensitivity (unable to measure incremental change, measured at inappropriate level).

c One indicator (12%) has adequate data source.

**Table 5:** Pilot Indicators and Data Sources for Heart Disease and Stroke Prevention, Health Care Setting, South Carolina and Alabama, 2001

**Indicator**		**Data Sources and Comments**	
1.	Percent of managed care organizations that adopt a policy to incorporate nationally accredited guidelines (e.g., the *AHA Guide to Primary Prevention of Cardiovascular Diseases* (17)) as part of their standard care package.^a^	1.	No data source found.
2.	Percent of managed care organizations that adopt a policy to incorporate nationally accredited guidelines (e.g., the *AHA Guide to Comprehensive Risk Reduction for Patients with Coronary and other Vascular Disease* (18)) as part of their standard care package.^a^	2.	No data source found.
3.	Percent of managed care organizations (e.g., health maintenance organizations, independent provider organizations, and preferred provider organizations) that have policies or guidelines to routinely provide or reimburse for assessments and counseling for physical activity, medical nutrition therapy, and tobacco cessation to plan members as part of their standard care package, according to the *Guide to Clinical Preventive Services* (19).^a^	3.	No data source found.
4.	Percent of health insurance plans that have policies or guidelines to routinely provide or reimburse for assessments and counseling for physical activity, medical nutrition therapy, and tobacco cessation to plan members as a covered benefit, according to the *Guide to Clinical Preventive Services* (19).^a^	4.	No data source found.
5.	Proportion of current and recent smokers who received advice to quit smoking from a health professional.	5.	Behavioral Risk Factor Surveillance System (BRFSS), optional Tobacco Indicators module (http://www.cdc.gov/brfss).^b^

a 4 indicators (80%) lack specificity (ambiguous, lack precision).

b 1 indicator (10%) has adequate data source.
